# Supplementary material for: Towards unified AI-driven fracture mechanics: the extended deep energy method (XDEM)
Source: Nat Commun. 2026 Aug 15;17:8492. doi: 10.1038/s41467-026-76748-1 (PMC13484471; doi:10.1038/s41467-026-76748-1)
Supplement: Supplementary file 3 [file 41467_2026_76748_MOESM3_ESM.zip › pic/Results/Stress_intensity_factor/Mode1/differ_a/crack_detailed_fields05.pdf]

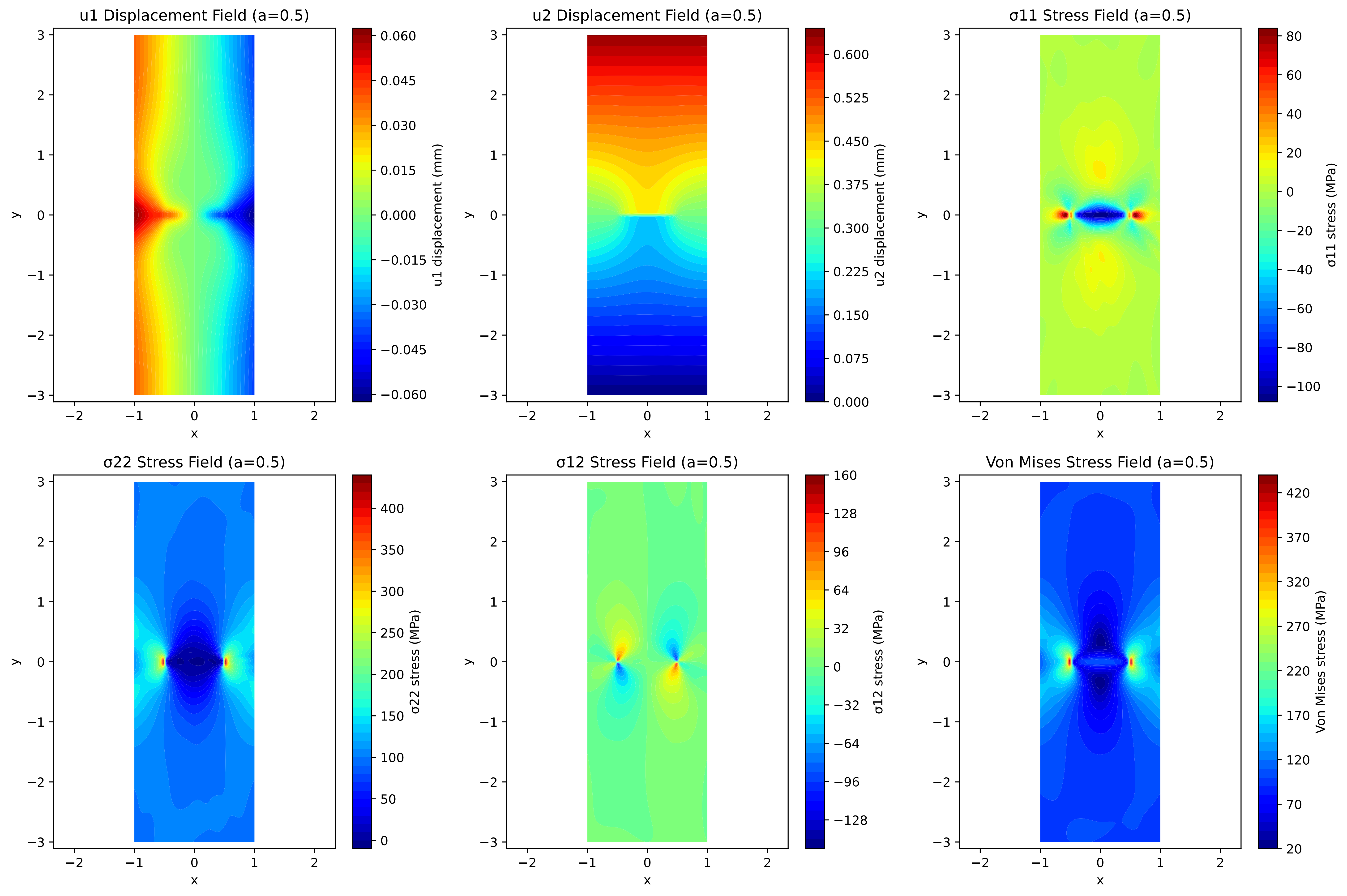
u1 Displacement Field (a=0.5)
u1 displacement (mm)
0.060
0.045
0.030
0.015
0.000
−0.015
−0.030
−0.045
−0.060
u2 Displacement Field (a=0.5)
u2 displacement (mm)
0.600
0.525
0.450
0.375
0.300
0.225
0.150
0.075
0.000
σ11 Stress Field (a=0.5)
σ11 stress (MPa)
80
60
40
20
0
−20
−40
−60
−80
−100
σ22 Stress Field (a=0.5)
σ22 stress (MPa)
400
350
300
250
200
150
100
50
0
σ12 Stress Field (a=0.5)
σ12 stress (MPa)
160
128
96
64
32
0
−32
−64
−96
−128
Von Mises Stress Field (a=0.5)
Von Mises stress (MPa)
420
370
320
270
220
170
120
70
20
x
y
−2
−1
0
1
2
3
−3
